# Effect of Ni^2+^, Zn^2+^, and Co^2+^ on green rust transformation to magnetite

**DOI:** 10.1186/s12932-022-00080-y

**Published:** 2022-12-29

**Authors:** Orion Farr, Evert J. Elzinga, Nathan Yee

**Affiliations:** 1 Department of Earth and Planetary Sciences, Rutgers University, Piscataway, NJ 08854 USA; 2 Department of Earth and Environmental Sciences, Rutgers University−Newark, Newark, NJ 07102 USA; 3 Department of Environmental Sciences, Rutgers University, New Brunswick, NJ 08901 USA

**Keywords:** Hydrosulfate green rust, Layered double hydroxides, Adsorption, Banded iron formations, Archean, Seawater, Nutrients, Diagenesis

## Abstract

In this study, we investigated Ni^2+^, Zn^2+^, and Co^2+^ mineralogical incorporation and its effect on green rust transformation to magnetite. Mineral transformation experiments were conducted by heating green rust suspensions at 85 °C in the presence of Ni^2+^, Zn^2+^, or Co^2+^ under strict anoxic conditions. Transmission electron microscopy and powder X-ray diffraction showed the conversion of hexagonal green rust platelets to fine grained cubic magnetite crystals. The addition of Ni^2+^, Zn^2+^, and Co^2+^ resulted in faster rates of mineral transformation. The conversion of green rust to magnetite was concurrent to significant increases in metal uptake, demonstrating a strong affinity for metal sorption/co-precipitation by magnetite. Dissolution ratio curves showed that Ni^2+^, Zn^2+^, and Co^2+^ cations were incorporated into the mineral structure during magnetite crystal growth. The results indicate that the transformation of green rust to magnetite is accelerated by metal impurities, and that magnetite is a highly effective scavenger of trace metals during mineral transformation. The implications for using diagenetic magnetite from green rust precursors as paleo-proxies of Precambrian ocean chemistry are discussed.

**Graphical Abstract:**

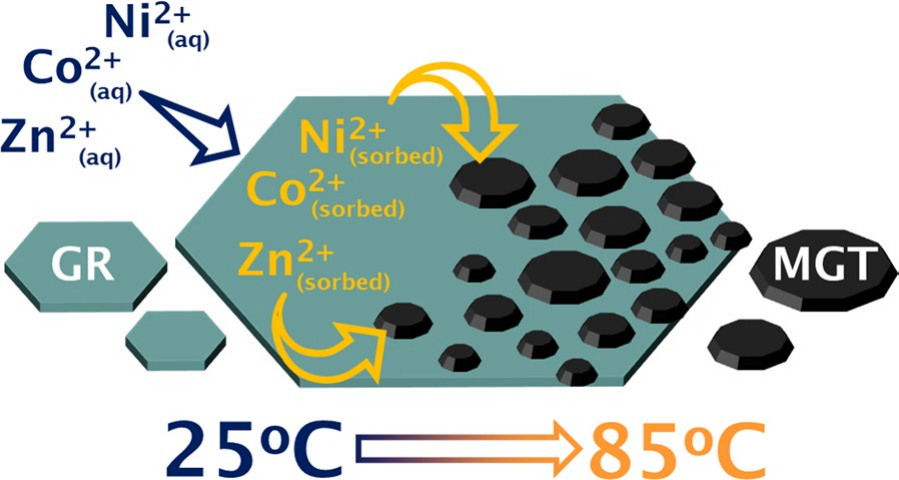

**Supplementary Information:**

The online version contains supplementary material available at 10.1186/s12932-022-00080-y.

## Introduction

Green rust (GR) minerals are a group of Fe^2+^/Fe^3+^layered double hydroxides (LDH) that are known to remove metal cations from solution. The GR crystal structure is composed of brucite-like octahedral sheets that envelop an interlayer of interchangeable anions (e.g. SO_4_^2−^, CO_3_^2−^, Cl^−^). The crystal edges contain reactive octahedral sites that are involved in metal binding [1] and divalent metal ions such as Ni^2+^ and Zn^2+^ are known to integrate into these flexibly charged surface sites [2–4]. Because GR minerals are commonly found in iron-rich sediments and anoxic waters [5–7] metal sorption by GR particles can play a significant role in contaminant sequestration and trace nutrient cycling [6–12].

Recently, green rust has been proposed as a mineral precursor of mineral assemblages preserved in Precambrian iron formations [13–15]. Green rust readily forms in Archean seawater-analogue solutions, and its precipitation in early oceans is thought to have been a major iron sink. GR has also been observed to precipitate in redox transition zones at Archean ocean analogue sites such as Lake Matano (Indonesia) and Arvadi Spring (Switzerland) [6, 7]. At Lake Matano, GR particles form near the iron redoxcline and persist through the water column at depth [6]. At Arvadi Spring, GR accumulates in sediments as flocs that cover the entire sediment surface [7]. Importantly, GR in these ferruginous environments have been found to sorb metal cations and control trace nutrient availability.

Under O_2_-free conditions, green rust can spontaneously convert to the more stable Fe^2+^/Fe^3+^iron oxide phase magnetite (Fe_3_O_4_) [16, 17]. The inverse spinel structure of magnetite is comprised of octahedral sites that are occupied by Fe^2+^ and Fe^3+^, and tetrahedral sites that only contain Fe^3+^. Because the magnetite structure has two times as much Fe^3+^, it has a lower Fe^2+^_/_Fe^3+^ratio compared to GR. The ideal chemical formula for GR sulfate is Fe_4_^2+^Fe_2_^3+^(OH)12SO_4_·8H_2_O [18] such that the Fe^2+^/Fe^3+^ ratio is 2, while the Fe^2+^/Fe^3+^ ratio for magnetite is 0.5. The anoxic conversion of GR to magnetite involves Fe^2+^ oxidation by water, where electrons transfer to aqueous H^+^ ions to produce H_2_ gas. The kinetics of GR transformation to magnetite is known to dependent on pH, Eh, and temperature [19]. However, little effort has been directed into elucidating the effect of trace metals on anoxic GR transformation even though metal sorption is known to modify GR surface charge and mineral reactivity [2, 3, 20–24]. So far, the effect of Ni^2+^, Zn^2+^, and Co^2+^ sorption on GR transformation to magnetite has not yet been studied.

Here we investigated Ni^2+^, Zn^2+^, and Co^2+^ mineralogical incorporation and its effect on GR conversion to magnetite. The objectives were: (1) to examine the sorption of Ni^2+^, Zn^2+^, and Co^2+^ metal ions during GR transformation to magnetite; (2) to determine the effect of trace metal incorporation on magnetite crystallization; and (3) to characterize the solid-phase distribution of metal cations in the magnetite transformation products. The work presented in this study provide new insights into metal sorption by magnetite during GR transformation, and the results have important implications for early Earth studies that use of magnetite as a paleo-proxy of Precambrian ocean chemistry.

## Materials and methods

### GR synthesis

Green rust sulfate (GR-SO_4_) was synthesized following the procedure described by Géhin et al. [25]. Briefly, a solution containing 150 mM FeSO_4_·7H_2_O and 25 mM Fe_2_(SO_4_)_3_ was prepared using deoxygenated ultra-purified water (milli-Q) and purged with N_2_ gas for 30 min in a sealed 125 mL reactor vial. A solution of 3 M NaOH was then injected into the reactor via a N_2_-purged needle and syringe and the mixture was gently shaken. A dark green precipitate formed in the reactor and powder X-ray diffraction analysis confirmed the precipitate was GR-SO4. The Fe concentration of this GR stock suspension was 200 mM and final pH was 6.8. The GR was stable at this pH and no transformation products were detected in the GR stock suspension during storage.

### Mineral transformation experiment

GR transformation experiments were conducted by heating GR suspensions at 85 °C under strict anoxic conditions. The temperature of 85 °C was selected to simulate the effects of GR burial and early diagenesis. Experiments were performed with GR suspensions in sealed serum bottles purged with N_2_ gas. GR suspensions were diluted with deoxygenated water to a final Fe concentration of 20 mM and the sealed serum bottles were then placed in an 85 °C water bath. At periodic intervals, the headspace gas and mineral suspension were sampled with needle and syringe. To remove O_2_, the needle and syringe were purged with N_2_ gas multiple times prior to sampling. The concentration of H_2_ in headspace samples was measured by gas chromatography (GC) using a Supelco GC column connected to a thermal conductivity detector (Model 310, SRI Instruments). GC was also used to analyze O_2_ to ensure that there was no atmospheric contamination. The pH was measured using a handheld HANNA pH probe in an anaerobic glove box.

To monitor mineral transformation, aliquots from the mineral suspension were collected at periodic intervals and the solids were analyzed using powder X-ray diffraction (XRD) and transmission electron microscopy (TEM). Samples were taken using N_2_-purged syringes and transferred to seal vials filled with nitrogen gas. For XRD analysis, the sample was centrifuged and the supernatant was removed. The solids were then dried with acetone under nitrogen flow. After adding a drop of glycerol to prevent oxidation, the solids were taken out of the vials using a spatula and transferred onto a glass plate for XRD analysis with a Rigaku MiniFlex 6G XRD equipped with a Co anode (λ = 1.790 Å). The X-ray source operated at 40 kV and 30 mA, scanning from 2 theta values of 1–90 degrees, at a scan speed of 1 degree per minute. For TEM analysis, 5 μL of sample from the vial was taken and the sample was taken deposited on a copper grid coated with carbon (Electron Microscopy Sciences CF-400-Cu) in an anaerobic glovebox (97% nitrogen, 3% hydrogen atmosphere). Excess solution was wicked away using filter paper and samples left to dry on the grid for several minutes. Grids were then transferred to a PELCO TEM Grid Vacuum Desiccator (PELCO Product # 16178) that was then pumped down using a vacuum to prevent sample oxidation. Images were captured using a Philip 420 Electron Microscope at 80 kV and analyzed using ImageJ.

### Metal incorporation experiment

Trace metal incorporation during the transformation of GR to magnetite was examined by adding a known amount of Ni, Zn or Co to GR suspensions prior to heating at 85 °C. Stock solutions of the metal ions were prepared by diluting 1000 ppm atomic absorption standards with milli-Q H_2_O and then titrating the solution with NaOH to a pH of 6.5. The metal solutions were deoxygenated by purging with N_2_ gas. Metal incorporation experiments were performed by adding either Ni, Zn or Co to anoxic GR suspensions ([Fe] = 20 mM) using N_2_-purged syringes. The final metal concentration (Ni, Zn or Co) was 1 ppm, corresponding to molar concentrations of 17 µM of Ni, 15 µM of Zn, and 17 µM of Co. These metal concentrations were selected because of their environmental relevance. The sealed serum bottles were gently shaken at room temperature for 2 h. One set of reactors were then transferred to an 85 °C water bath and heated to induce mineral transformation for up to 24 h. Another set of reactors were kept at room temperature as controls. Throughout the first 2 h and during the 85 °C heating period, aliquots of the mineral suspension were collected for analysis. Mineral transformation was tracked using XRD measurements and the dissolved metal concentrations were analyzed using inductively coupled plasma optical emission spectroscopy (ICP-OES). XRD was performed using the methods described above. To determine the dissolved metal concentrations, samples were filtered using 0.2 µm nylon filters into test tubes containing 2% HNO_3_. The metal concentrations in the acidified filtrates were analyzed using an ICPAP-7400 ICP-OES Duo. The concentrations of Ni, Zn, Co, and Fe were measured at 231.6 nm, 213.9 nm, 228.6 nm, and 261.2 nm wavelengths, respectively.

At the end of each transformation experiment, an acid dissolution assay was performed to examine the distribution of the incorporated metals. The magnetite product formed during heating was collected by centrifugation (2000 rpm, 5 min), and dried with flowing N_2_ gas for 30 min. The solids were then mixed with 4 M HCl to dissolve the magnetite product and aliquots were sampled at periodic intervals to monitor mineral dissolution and metal release. Complete dissolution of the solids occurred within 1 h of reaction with the acid. Samples were filtered (0.2 µm) and dissolved metal concentrations (Fe, Ni, Zn, Co) were measured using ICP-OES.

## Results

**Fig. 1:**
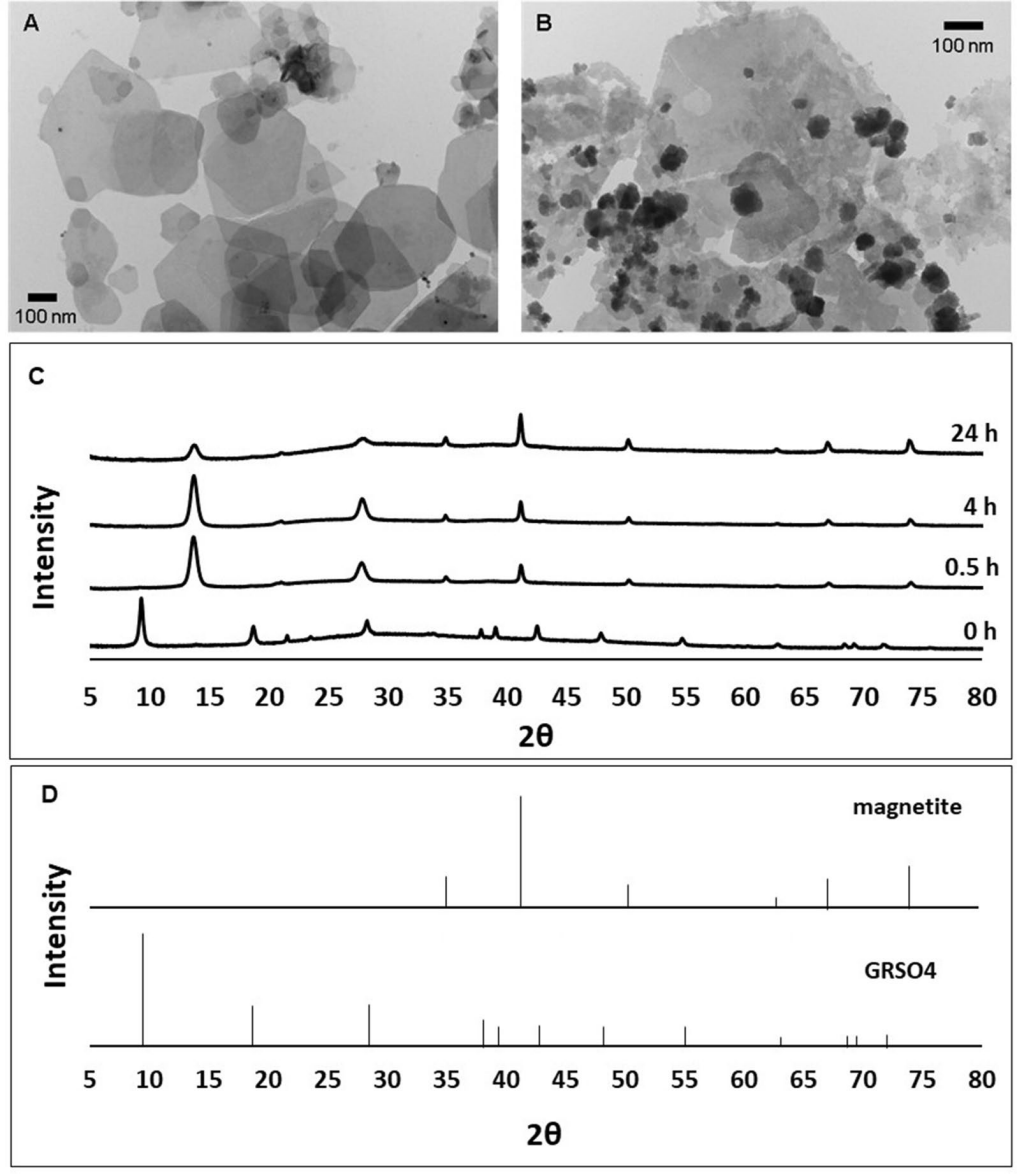
Green rust transformation to magnetite. **A** TEM image of GR hexagonal platelets before transformation; **B** TEM image cubic magnetite crystals after transformation; **C** X-ray diffractograms of GR samples heated at 85° C under anoxic conditions; **D** Reference X-ray diffractograms of magnetite and green rust sulfate

Heating GR suspensions at 85 °C under anoxic conditions resulted in the production of H_2_ gas (Additional file 1: Fig. S1),, a decrease in mineral volume, and the precipitation of a dark magnetic product (Additional file 1: Fig. S2). The solution pH dropped slowly and continuously from pH 6.8 to 6.1 over the 24 h experiment (Additional file 1: Fig. S3). Transmission electron micrographs of the parent GR material showed hexagonal platelets approximately 500 nm in diameter (Fig. 1a), and transformation of this GR material resulted in the formation of electron dense cubic crystals 20–30 nm in size (Fig. 1b). XRD analysis of the transformation products indicate that GR was converted to magnetite (Fig. 1c). The starting material displayed XRD peaks at 9.3° and 18.7° 2-theta angles, corresponding to d-spacings of 11.05 Å and 5.51 Å of the layered double hydroxide GR structure. After heating of the GR suspensions for 30 min, a single interlayer GR reflection 13.67° 2-theta corresponding to a d-spacing of 7.52 Å was observed. The appearance of the single interlayer GR reflection and the disappearance of the original interlayers spacings indicate contraction of the hydroxide sheets caused by the expulsion of interlayer SO_4_^2−^ oxyanions. This modification of the GR structure was concurrent with the appearance of 2-theta peaks at 41.20°, 34.92 ^o^, 50.24 ^o^, 67.03 ^o^ and 73.85 ^o^ corresponding to the d-spacings 2.54 Å, 2.98 Å, 2.11 Å, 1.62 Å, 1.49 Å, respectively, indicating the formation of magnetite. As the reaction progressed, the XRD peak intensities of magnetite increased and those of GR decreased. Both TEM and XRD analyses showed a mixture of GR and magnetite at the end of the 24 h experiment, indicating the presence of residual GR and incomplete mineral transformation. Control experiments conducted at room temperature showed no H_2_ production or change in pH, and the GR remained stable with no mineral transformation occurring for over 1 year.

**Fig. 2:**
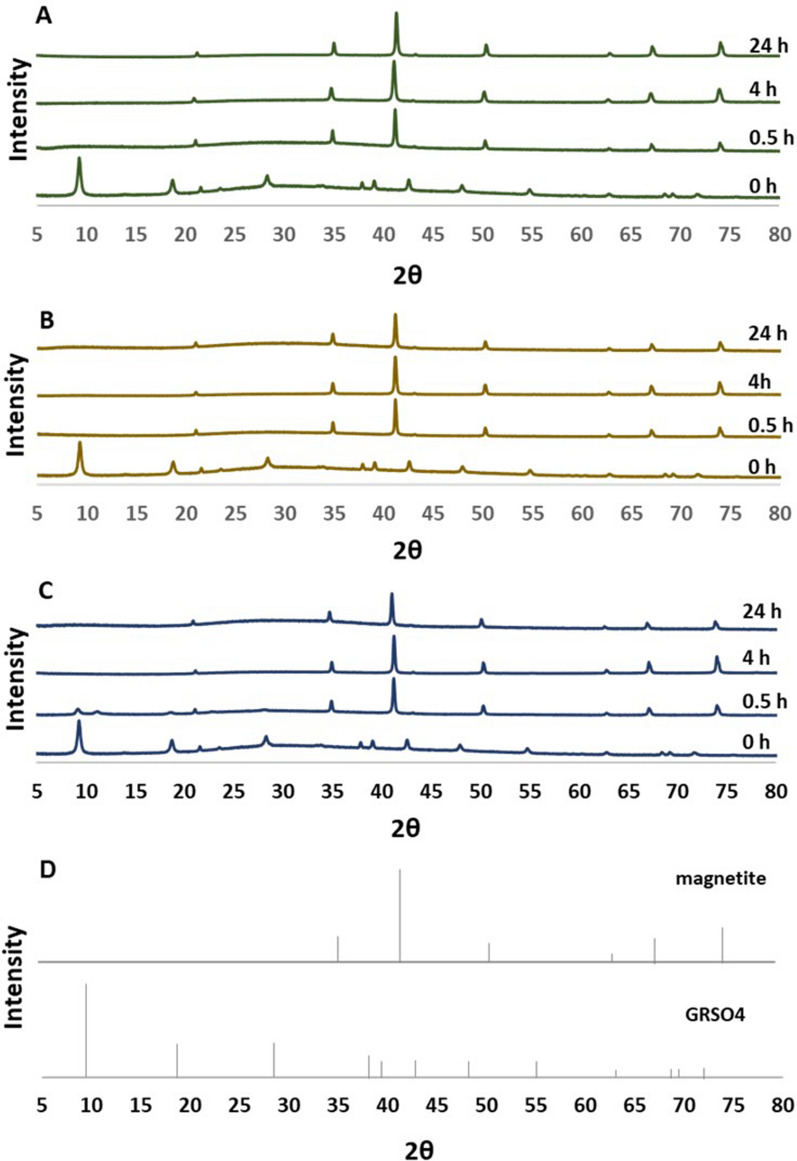
GR transformation to magnetite in the presence of metal cations. GR suspensions were heated at 85 °C under anoxic conditions in the presence of 1 ppm **A** nickel; **B** zinc or **C** cobalt. **D** Reference X-ray diffractograms of magnetite and green rust sulfate

The addition of metal cations accelerated the transformation of GR to magnetite (Fig. 2). When 1 ppm of either Ni^2+^ and Zn^2+^ was added to the GR suspension, complete transformation to magnetite was observed by 0.5 h of heating at 85 °C, while the addition of 1 ppm Co^2+^ resulted in complete GR transformation in less than 2 h. Residual GR was not detectable by XRD in any of the metal-mineral systems at the end of the 24 h experiments.

**Fig. 3:**
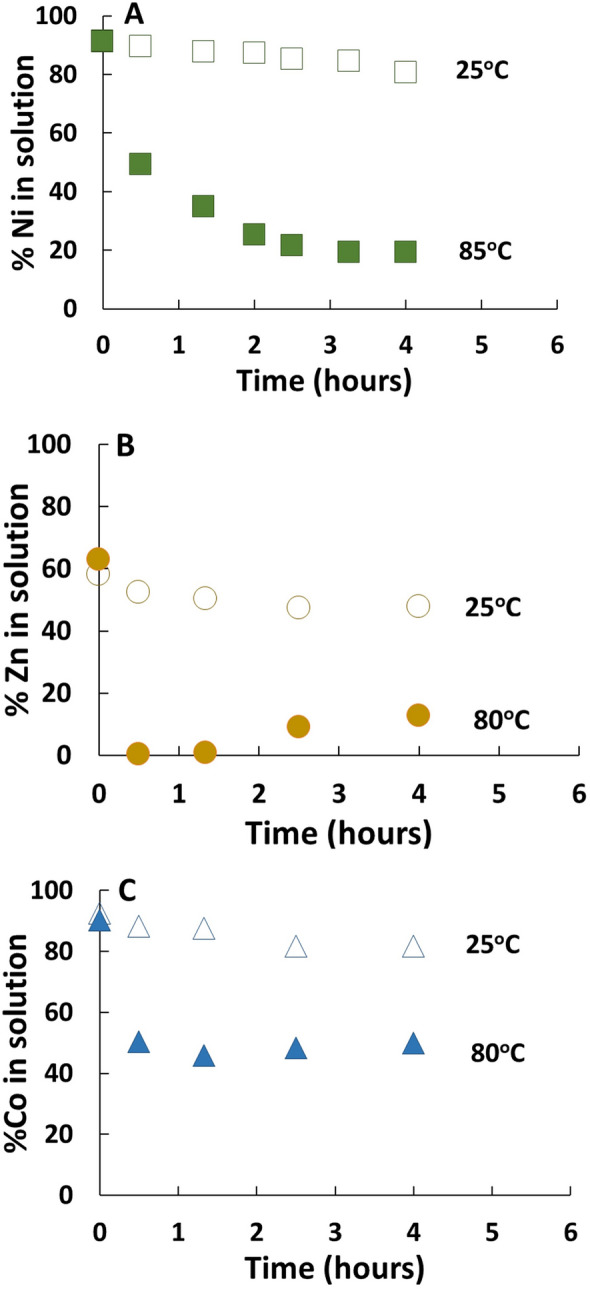
Sorption of metal cations during GR transformation to magnetite. GR suspensions were pre-equilibrated with either Ni^2+^, Zn,^2+^, or Co^2+^ for 2 h and then transferred to a 85 °C water bath. Solid symbols represent the fraction of dissolved metal remaining in solution during mineral transformation at 85 °C. Open symbols represent control experiments conducted at room temperature. Experiments were carried out with the metal cations **A** nickel, **B** zinc, and **C** cobalt

Chemical analyses of the aqueous solutions showed that the conversion of GR to magnetite resulted in the rapid removal of dissolved Ni^2+^, Zn^2+^, and Co^2+^ (Fig. 3), indicating that the metal cations were sorbed/co-precipitated with magnetite during mineral transformation. In the Ni^2+^ experiment, pre-equilibration of the metal solution with GR at room temperature led to sorption of approximately 9% of total Ni^2+^_(aq)_ (Fig. 3a). Upon heating, Ni^2+^ was quickly partitioned to the solid phase with over 85% of Ni^2+^_(aq)_ removed after 4 h of reaction. Replicate experiments exhibited excellent agreement between independent experimental runs (Additional file 1: Fig. S4). A longer experiment conducted up to 24 h showed a small amount (< 10%) of Ni re-released back into the aqueous solution (Additional file 1: Fig. S5). The room temperature control experiment showed slow continuous Ni^2+^_(aq)_ sorption on GR with approximately 20% of Ni^2+^_(aq)_ removed after 4 h and 28% removed after 24 h. In the Zn^2+^ experiment, high affinity sorption of Zn^2+^ into the solid phase was observed (Fig. 3b). Approximately 40% of the Zn^2+^_(aq)_ was sorbed to GR in the pre-equilibration period. The conversion of GR to magnetite resulted in complete Zn^2+^_(aq)_ removal at the first sampling time point (30 min), followed by low levels Zn^2+^ release back to solution. After 4 h, 87% of the Zn^2+^_(aq)_ was removed at 85 °C heating compared to 52% at room temperature. In the Co^2+^ experiment, lower affinity sorption of Co^2+^ onto the solid phase was observed. Pre-equilibration of the Co^2+^ solution with GR at room temperature removed 10% of the Co^2+^_(aq)_. Heating at 85 °C resulted in 50% of Co^2+^_(aq)_ removal at 30 min, with no additional Co^2+^ removal detected at the following time points. Similar to the other metals, slow continuous Co^2+^ removal by GR was observed in the room temperature control experiments with approximately 18% of Co^2+^ sorption after 4 h. Finally, decreases in solution pH were observed during GR conversion to magnetite (Additional file 1: Fig. S6), and the changes in pH (6.8 to 6.2) were nearly identical to the metal-free control experiment (Additional file 1: Fig. S3).

**Fig. 4:**
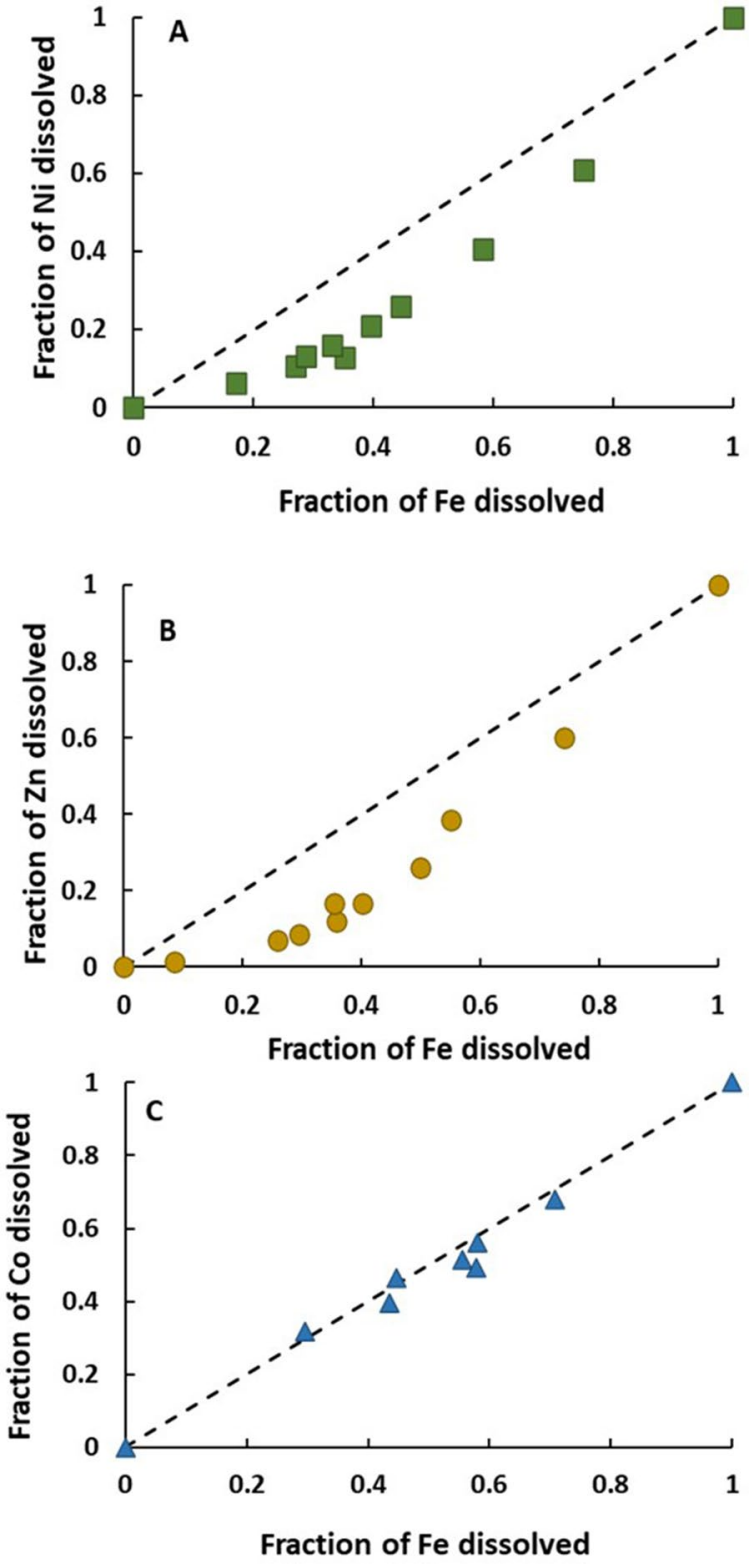
Dissolution ratio curves. The fraction of metal cations released with the fraction of dissolved Fe from the magnetite product. The square, circle, and triangle symbols represent the dissolution of **A** nickel; **B** zinc and **C** cobalt, respectively. The dashed line represents the 1:1 dissolution curve

Dissolution of the magnetite product with HCl yielded dissolution ratio curves that revealed the distribution of metal cations within the magnetite structure (Fig. 4). The dissolution ratio curves of Ni to Fe and Zn to Fe showed concave trends, where Fe was preferentially released over the metal cations when magnetite was dissolved. These data indicated that the metal cations were predominately integrated into the interior of the magnetite lattice. Conversely, the dissolution ratio curve of Co to Fe yielded a straight line with a slope close to 1. This indicated that Co was uniformly distributed in the magnetite structure. None of the metal ions exhibited a convex dissolution trend, excluding surface adsorption as a major mechanism.

## Discussion

The primary finding of the work presented here is that the transformation of GR to magnetite is markedly accelerated in the presence of metal impurities. This suggests that the interaction of trace metal sorbates with GR goes beyond simple surface complexation reactions and involves reactions that destabilize the GR structure. We propose that the three metal cations (Ni^2+^, Zn^2+^, and Co^2+^) replace structural Fe^2+^ cations at GR crystal edge sites and/or in the near-surface lattice, consistent with the results of Elzinga [4]. The ability of these metals to replace Fe^2+^ can be attributed to their identical valence and similar ionic radii facilitating isomorphous substitution. Exchange may be promoted by the higher hydrolysis constants of the three metals relative to Fe^2+^ (Zn^2+^  > Ni^2+^  > Co^2+^  > Fe^2+^), as more hydrolyzable cations are more readily adsorbed and coprecipitated [3]. Substitution may further be enhanced by GR dissolution-reprecipitation reactions driven by solubility differences between the added metals and Fe^2+^, or by Fe^2+^-catalyzed GR recrystallization. The resultant substitution of metals into the GR lattice at and near particle edges is a plausible explanation for the observed decrease in GR stability. The incorporation of metal impurities is expected to weaken the GR structure, making the mineral less stable and more susceptible to transformation relative to the pure phase. This explanation also agrees with previous observations of decreased GR mineral stability and increased susceptibility towards mineral transformation due to divalent cation sorption [3, 24, 26, 27].

The XRD results presented in Fig. 2 suggest that GR is rapidly transformed to magnetite, whereas the metal sorption kinetics curves in Fig. 3 exhibit longer term dynamics. Because XRD can only identify crystalline phases, poorly crystalline materials such as degrading GR during the early stages of magnetite nucleation may have evaded detection [28]. It is therefore conceivable that the metal sorption kinetics were controlled by GR dissolution and concurrent magnetite neoformation that were ongoing even after the disappearance of the GR peaks from the diffractograms.

The conversion of GR to magnetite was marked by a significant increase in Ni^2+^ Zn^2+^ and Co^2+^ cation uptake from solution, demonstrating a stronger affinity for metal sorption/co-precipitation of the magnetite product compared to the GR precursor. Increased metal sorption can be attributed to changes in mineral surface charge and surface area during transformation. The pH_zpc_ of magnetite is 6–6.8 [29] compared to the pH_zpc_ of GR of 8.3 [30]. In the pH range of the experiment (pH 6.8 to 6.1), magnetite is expected to exhibit a net negative surface charge which would strongly favor cation adsorption. Furthermore, the particle size of the magnetite product was considerably smaller than the GR precursor resulting in higher reactive surface area (Fig. 1). The rapid nucleation of magnetite would also promote small crystal formation leading to high surface area in the initial stages of magnetite precipitation.

The adsorption of metal cations to magnetite surfaces during crystal growth resulted in structural incorporation of metals into the mineral lattice (Fig. 4). The congruency plots for Ni and Zn showed preferential accumulation of the metal cations in the interior of the magnetite crystals. This is likely due to a combination of GR dissolution and magnetite crystal growth processes. The removal of Ni^2+^_(aq)_ and Zn^2+^_(aq)_ by GR during the pre-equilibration period was driven by sorption reactions at the edges of GR particles where the lattice is disrupted, and not along the chemically stable basal planes [4, 31]. Because of their chemical reactivity, the edge sites are also expected to be the location where the oxidation reactions transforming GR to magnetite take place [32]. The early-stage magnetite crystals therefore likely incorporated the Ni^2+^ and Zn^2+^ that had accumulated at the GR edges during pre-equilibration, resulting in elevated impurity contents. Furthermore, there were higher levels of aqueous Ni^2+^ and Zn^2+^ available for incorporation in the early stages of magnetite crystallization compared to the later stages (Fig. 2). This indicates that the nuclei of the early-stage magnetite crystals sequestered high levels of aqueous metal cations into the core of the mineral during neoformation, leaving behind lower concentrations of dissolved Ni^2+^ and Zn^2+^ at the later-stages of crystal growth.

The even distribution of Co^2+^ suggests a different mechanism where cobalt incorporation is limited not by its concentration but by some other factor that scales with the rate of magnetite crystal growth. One possibility is that oxidation of Co^2+^ to Co^3+^ occurred during magnetite precipitation and cobalt incorporation, and that concurrent Co^2+^ and Fe^2+^ oxidation resulted in the uniform incorporation of cobalt as Co^3+^ into the magnetite lattice. The potential for Co^2+^ oxidation to Co^3+^ during magnetite formation has been previously reported [33, 34]. Co^2+^ oxidation and the incorporation of Co^3+^ may be linked to slower kinetics of GR dissolution in the cobalt experiment (Fig. 2C). Additional studies addressing the speciation of the incorporated metals in the magnetite structure are warranted to further elucidate the mechanism of metal integration.

### Geochemical implications

The results of this study indicate that Ni^2+^, Zn^2+^ and Co^2+^ sorption is coupled to GR transformation to magnetite. Because GR is a precursor to magnetite found in sedimentary rocks [13–15, 35] our findings have important implications for understanding the trace metal composition of iron oxides preserved in the geologic record. Field studies have shown that GR is one of the dominant Fe minerals that deposit in anoxic sediments at Archean analogue sites [6, 7]. Our experiments indicate that increases in temperature during sediment diagenesis can trigger GR transformation to magnetite. The mineral conversion process, which likely occurs during early diagenesis, is a key step in metal transfer to magnetite crystals. This is a significant insight because trace metal composition of ancient magnetite-containing rocks has been used to infer the aqueous metal concentrations of Archean oceans [36–39]. If iron formations are to be used as chemical archives of paleo-seawater, then it is crucial to understand the relative roles of GR and magnetite in metal sorption, as well as the geochemical conditions that control metal transfer to the mineral phase. Our results demonstrate that the reactivity of GR differs from that of magnetite, and that magnetite is a more effective scavenger of metals, including Ni^2+^ a metal which is thought to be an essential micronutrient for methanogenesis. Furthermore, mineral transformation reactions that occurs after burial imply that the metal impurities found in magnetite reflect conditions during diagenesis and sediment pore water composition rather than overlying water column chemistry. Based on these findings, caution should be exercised when using diagenetic magnetite as a paleo-proxy for nutrient availability in Precambrian oceans.

## Supplementary Information

**Additional file 1: Figure S1.** Production of H_2_ gas. GR suspensions were heated at 85^o^C under anoxic conditions and headspace was analyzed using gas chromatography. **Figure S2.** Formation of a magnetic product. GR samples were heated at 85^o^C for 24 hours. The starting GR material is shown on the right and the magnetic product is shown on the left. **Figure S3.** Change in solution pH during GR transformation. Open symbols represent the room temperature control experiment and the closed symbol represent GR heated at 85^o^C. **Figure S4.** Replicate experiments of Ni incorporation during GR transformation to magnetite. Open symbols represent the room temperature control experiment, and the closed symbols represent 3 independent experiments of GR heated at 85^o^C in presence of 1 ppm Ni. **Figure S5.** 24 hour experiment of Ni incorporation during GR transformation to magnetite. Open symbols represent the room temperature control experiment and the closed symbols represent GR heated at 85^o^C. **Figure S6.** Changes in solution pH during GR transformation in the metal amended experiments. Open symbols represent the room temperature control experiment and the closed symbols represent GR samples heated at 85^o^C containing A) nickel, B) zinc, and C) cobalt.

## Data Availability

The datasets used for this manuscript are displayed in the figures in the manuscript and the additional file. The data in tabulated form are available upon request.
