# Supplementary material for: Effect of Ni2+, Zn2+, and Co2+ on green rust transformation to magnetite
Source: Geochem Trans. 2022 Dec 29;23:3. doi: 10.1186/s12932-022-00080-y (PMC9798576; doi:10.1186/s12932-022-00080-y)
Supplement: Supplementary file 1 — Additional file 1: Figure S1. Production of H2 gas. GR suspensions were heated at 85oC under anoxic conditions and headspace was analyzed using gas chromatography. Figure S2. Formation of a magnetic product. GR samples were heated at 85oC for 24 hours. The starting GR material is shown on the right and the magnetic product is shown on the left. Figure S3. Change in solution pH during GR transformation. Open symbols represent the room temperature control experiment and the closed symbol represent GR heated at 85oC. Figure S4. Replicate experiments of Ni incorporation during GR transformation to magnetite. Open symbols represent the room temperature control experiment, and the closed symbols represent 3 independent experiments of GR heated at 85oC in presence of 1 ppm Ni. Figure S5. 24 hour experiment of Ni incorporation during GR transformation to magnetite. Open symbols represent the room temperature control experiment and the closed symbols represent GR heated at 85oC. Figure S6. Changes in solution pH during GR transformation in the metal amended experiments. Open symbols represent the room temperature control experiment and the closed symbols represent GR samples heated at 85oC containing A) nickel, B) zinc, and C) cobalt. [file 12932_2022_80_MOESM1_ESM.docx]

**Additional files**

**Effect of Ni^2+^, Zn^2+^, and Co^2+^ on Green Rust Transformation to Magnetite**

Orion Farr^a^, Evert J. Elzinga^b^, Nathan Yee^a,c^*

^a^Department of Earth and Planetary Sciences Rutgers University, Piscataway, NJ 08854, USA

^b^Department of Earth and Environmental Sciences, Rutgers University−Newark, Newark, NJ 07102, USA

^c^Department of Environmental Sciences, Rutgers University, New Brunswick, NJ 08901, USA

*Corresponding author: nyee@envsci.rutgers.edu

**Figure S1.** Production of H_2_ gas. GR suspensions were heated at 85^o^C under anoxic conditions and headspace was analyzed using gas chromatography.


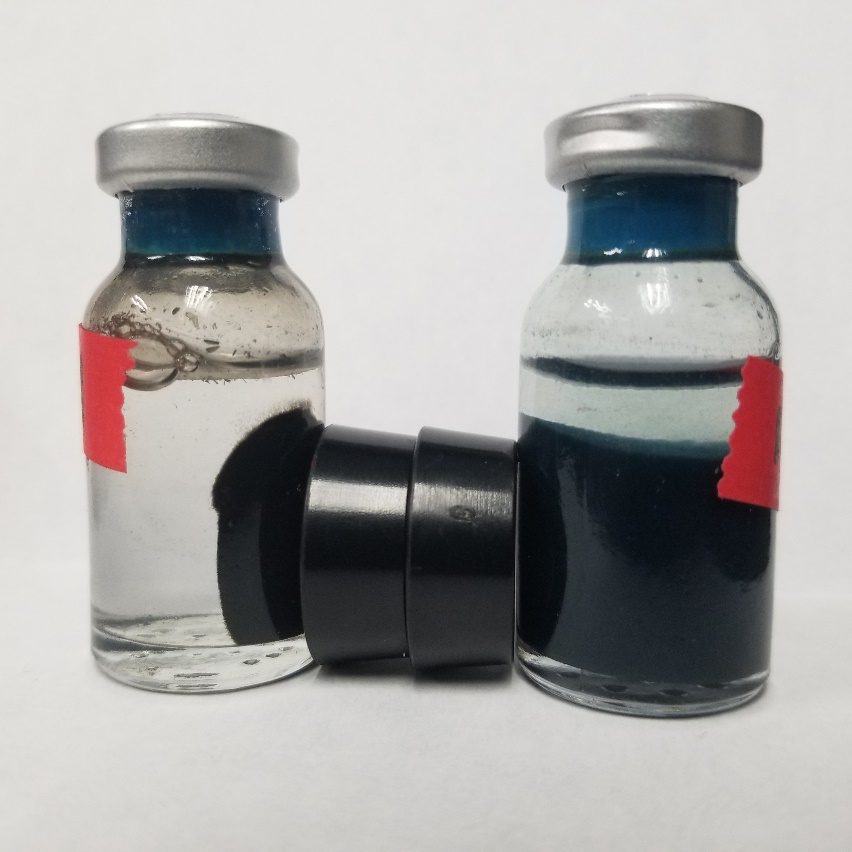


**Figure S2.** Formation of a magnetic product. GR samples were heated at 85^o^C for 24 hours. The starting GR material is shown on the right and the magnetic product is shown on the left.

**
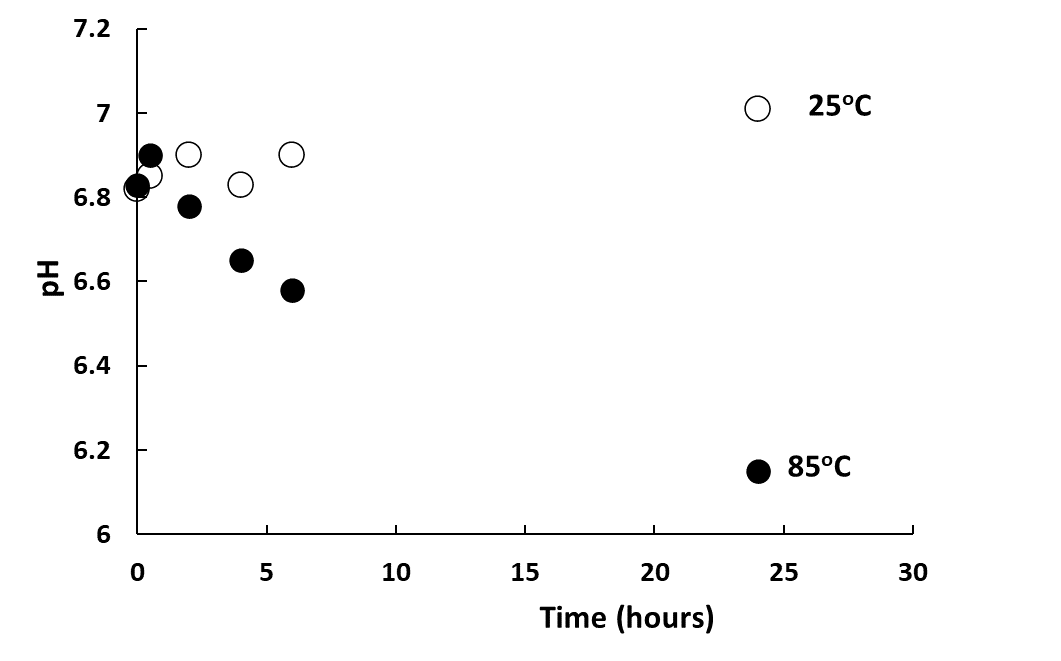
**

**Figure S3.** Change in solution pH during GR transformation. Open symbols represent the room temperature control experiment and the closed symbol represent GR heated at 85^o^C.

**Figure S4.** Replicate experiments of Ni incorporation during GR transformation to magnetite. Open symbols represent the room temperature control experiment, and the closed symbols represent 3 independent experiments of GR heated at 85^o^C in presence of 1 ppm Ni.

**Figure S5.** 24 hour experiment of Ni incorporation during GR transformation to magnetite. Open symbols represent the room temperature control experiment and the closed symbols represent GR heated at 85^o^C.

**
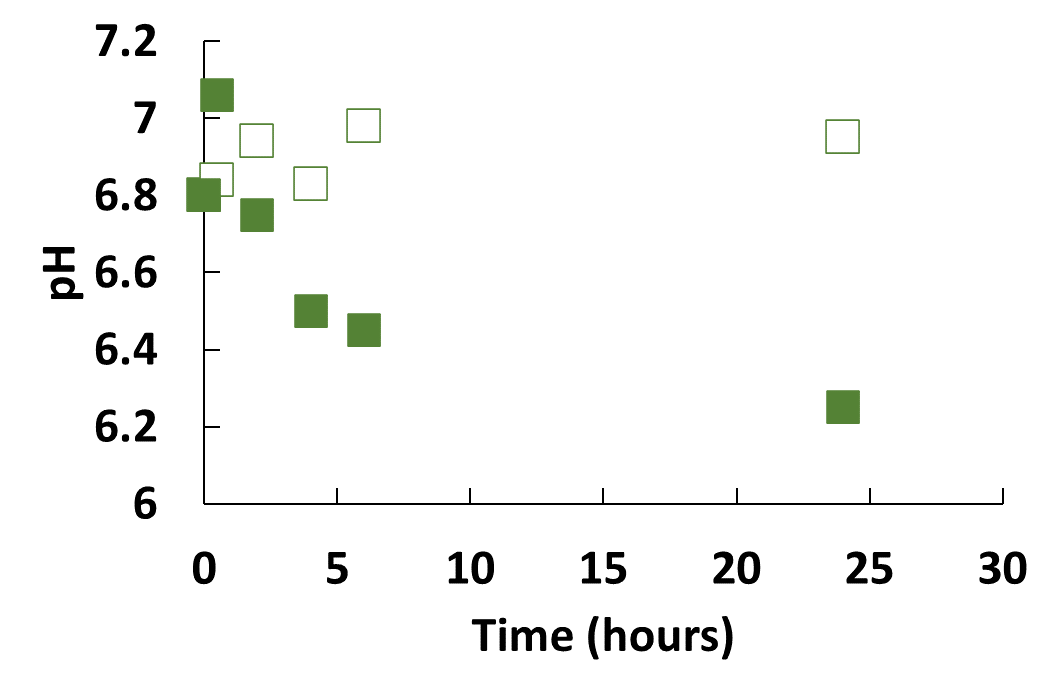
**

**A**

**
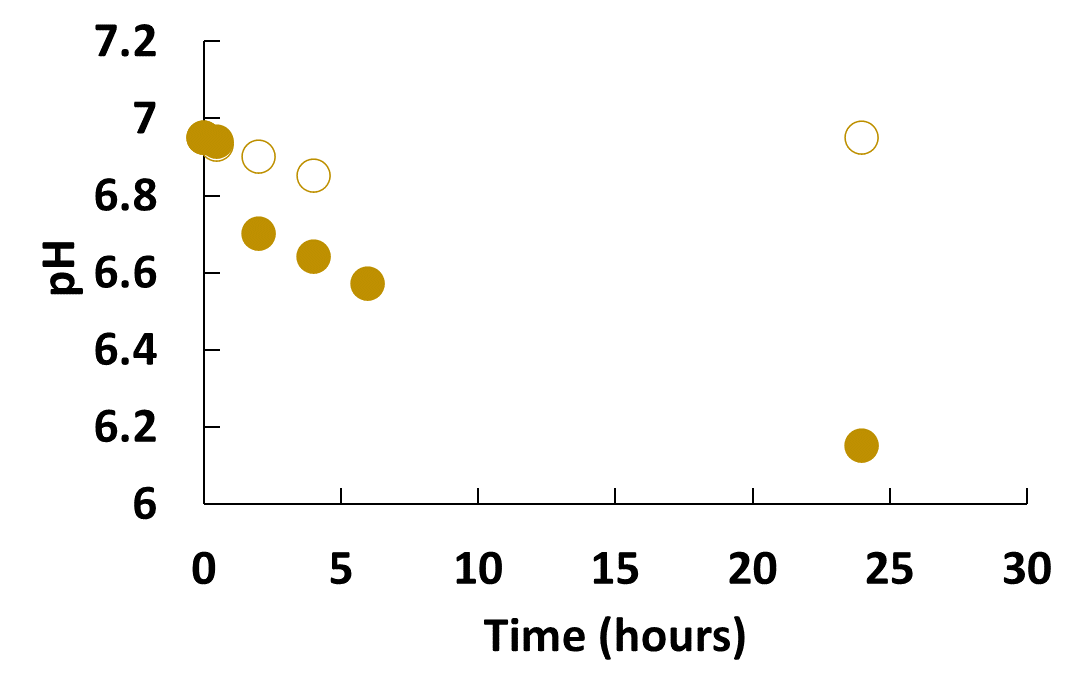
**

**B**

**C**

**
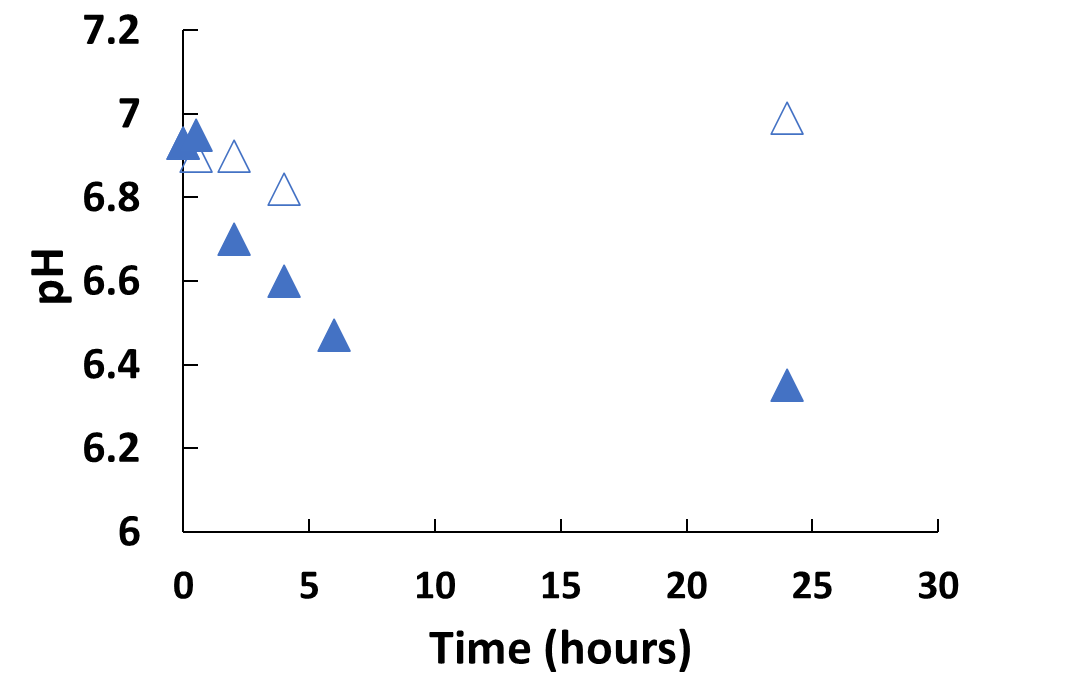
**

**Figure S6.** Changes in solution pH during GR transformation in the metal amended experiments. Open symbols represent the room temperature control experiment and the closed symbols represent GR samples heated at 85^o^C containing A) nickel, B) zinc, and C) cobalt.
